# Involuntary Treatments in Italy: a Debated Issue

**DOI:** 10.1192/j.eurpsy.2022.131

**Published:** 2022-09-01

**Authors:** B. Carpiniello

**Affiliations:** Department of Medical Sciences and Public Health University of Cagliari, Italian Psychiatric Association; Secretary Of The Epa-council Of Npas, Cagliari, Italy

**Keywords:** involuntaty treatments add restraint add Italy

## Abstract

Involuntary treatments probably are the most critical issue for psychiatric practice all over the world, including Italy, where the public debate about involuntary admissions and related coercive measures has been constantly alive. In Italy involuntary treatments are justified on the basis of three criteria: the presence of a mental illness; the need for urgent hospital-based treatment, the patient refusal of treatment. Although only 10% of all hospitalizations in Italy occur on a involuntary basis, actually the lowest rate in Europe, proposals of modification of the current Law have been repeatedly presented, in terms of further restrictions of the conditions allowing involuntary hospitalization or even in terms of its abolition. The practice of physical restraint in particular, which has been reported as applied in approx. 85% of Psychiatric Wards, has been strongly criticized, although the effective dimension of its use in Italy is unknown due the lack of official data. In 2015 The National Council of Bioethics expressed a series of doubts and criticisms as well as the Special Commission for Human Rights of the Italian Senate in 2016. Moreover, the death of some patients submitted to physical restraint in the last years, gave repeteadly rise to a media hype, leading again very recently to claims for the abolition of any form of physical restraint during a National Conference on Mental Health, a proposal that the Minister of Health welcomed, committing himself to implement it through a agreement between the State and the Regions, officially devoted to health assistance in Italy.

**Disclosure:**

No significant relationships.

